# 1 Mb Deletion in 10q26.3 and the Likely Pathogenic Variant in the TRIO Gene: A Twin Case Study Challenging Their Role in Autism Diagnosis

**DOI:** 10.1155/crpe/8859738

**Published:** 2025-09-15

**Authors:** Silvia Lakatošová, Michaela Miklošovičová, Michal Konečný, Lenka Wachsmannová, Gabriela Krasňanská, Mária Kopčíková, Petra Keményová, Miroslav Tomka, Jana Lisyová, Daniela Ostatníková, Gabriela Repiská

**Affiliations:** ^1^Institute of Physiology, Academic Research Center for Autism, Faculty of Medicine, Comenius University in Bratislava, Bratislava, Slovakia; ^2^Ambulance of Clinical Genetics, Department of Clinical Genetics, Institute of Medical Biology, Genetics and Clinical Genetics, Faculty of Medicine, Comenius University in Bratislava and University Hospital, Bratislava, Slovakia; ^3^Laboratory of Genomic Medicine, GHC GENETICS SK, s.r.o., Bratislava, Slovakia; ^4^Institute of Biology and Biotechnology, Department of Biology, Faculty of Natural Sciences, University of St. Cyril and Methodius in Trnava, Trnava, Slovakia; ^5^Institute of Medical Biology, Genetics and Clinical Genetics, Faculty of Medicine, Comenius University, Bratislava, Slovakia; ^6^Department of Molecular and Biochemical Genetics, Institute of Medical Biology, Genetics and Clinical Genetics, Faculty of Medicine, Comenius University in Bratislava and University Hospital, Bratislava, Slovakia

## Abstract

Here, we present a case study of twin boys aged 2 and 7 years who both met the diagnostic criteria for autism spectrum disorders (ASDs) based on the standard diagnostic instruments ADOS-2 and ADI-R. The clinical indication for genetic diagnostics in the first boy was autism with high severity of symptoms, delayed speech development, and mild facial dysmorphia. The second boy's indication was autism with moderate severity of symptoms, delayed speech development, mild facial features, slowed psychomotor development, and microcephaly. The microarray-based analysis of chromosome aberrations revealed a heterozygous 977,456 bp deletion of region 10q26.3 in both boys. The region includes 28 genes, some of these genes are important in the development of the central nervous and urogenital systems, and heterozygous deletions in this region have been associated with mental retardation, growth and development disorders, and craniofacial anomalies. The whole exome sequencing confirmed the presence of this deletion in both boys and, at the same time, led to the identification of a pathogenic SNV variant in the TRIO gene in the boy with microcephaly and delayed psychomotor development, which may explain the different phenotype of both boys. However, the segregation analysis of these variants in the family revealed that the microdeletion was inherited from the asymptomatic father, and the c.2149C > T variant in the TRIO gene was inherited from the asymptomatic mother, making the diagnostic finding uncertain. This case highlights that when pathogenic or likely pathogenic variants are inherited from unaffected parents, the clinical phenotype may result from a combined burden of multiple rare variants and polygenic risk, underscoring the importance of a comprehensive genomic analysis in complex cases. Thus, we emphasize the importance of utilizing available methods, such as whole exome sequencing besides microarray-based comparative genomic hybridization, in the genetic diagnosis of autism patients in Slovakia.

## 1. Introduction

Autism spectrum disorder (ASD) is a neurodevelopmental disorder with high heritability estimates. The DNA diagnostic procedure struggles in an attempt to identify causal DNA variants, mainly due to the polygenic architecture, incomplete penetrance, and common genetic variation that contribute to the manifestation of the disorder [1]. Up-to-date DNA diagnostic procedures include copy-number variation analyses by microarray-based methods and whole exome or genome sequencing procedures. Approximately 25% of ASD cases belong to the group of molecularly defined autism, where a causal single nucleotide variant, structural, or copy-number variant is identified, including ASD cases associated with a known genetic syndrome [2]. The remaining 75% of ASD cases remain currently undefined, potentially due to the polygenic architecture. Here, we present a case study of twin boys with autism diagnosed at the Academic Center for autism research in Bratislava, Slovakia. The present study was approved by the Ethics Committee of the Faculty of Medicine of Comenius University and the University Hospital in Bratislava, Slovakia, in accordance with the 1964 Declaration of Helsinki and its subsequent amendments. The parents of twin boys were informed of the study design, and they signed the written informed consent. The study is blinded, and no personal identification is possible, and the initials of the probands are fabricated.

## 2. Case Presentation

Twin boys came for examination at the age of 2 years and 7 months. They were dizygotic diamniotic dichorionic twins from a high-risk pregnancy. The clinical indication for genetic examination included autism, delayed speech development, and mild facial dysmorphia in the first boy (A.V.). The boy had a negative perinatal history, and he was rehabilitated for hypertonia. His motor development was within the broader norm. The current neurological findings were physiological, and the USG of the brain was normal. He had delayed speech development, he spoke his first words at the age of 18 months, and then he stopped talking. ASD diagnosis based on the standard diagnostic instruments Autism diagnostic Interview–revised (ADI-R) and Autism Diagnostic Observation Schedule (ADOS-2) [3, 4] pointed out the high severity of all autism symptoms (comparative severity scores for social affect, restrictive and repetitive behavior, and overall comparative severity scores were 9, 10, and 10, respectively). In the objective findings, he had normocephaly (head circumference: 47 cm) and nonspecific dysmorphic features, such as eye slits slanted laterocaudally, deeper set bulbs, wider nasal root, longer philtrum, and transverse groove on the right palm.

His brother's (N.V.) clinical indication included autism, delayed psychomotor development, microcephaly, and facial dysmorphia. He had fetal hypotrophy and was born with low birth weight, for which he was monitored at the intensive care unit. He was rehabilitated for hypertonia. His motor development was within the broader norm. The current neurological findings were physiological, and USG of the brain showed a mild ventriculomegaly, the state after bilateral intraventricular hemorrhage (IVH I-II) in the norm. He had delayed speech development and anger affects. The diagnosis of ASD was confirmed by ADI-R and ADOS-2 instruments with moderate symptom severity (comparative severity scores for social affect, restrictive and repetitive behavior, and overall comparative severity scores were 9, 6, and 7, respectively). His intellect and cognitive abilities were not assessed; however, the neurologist suspected an intellectual developmental delay only in N.V. In the objective findings, he had microcephaly with occipital plagiocephaly (head circumference: 45.5 cm, −2.83 SD), asthenic habitus, and nonspecific dysmorphic features, such as slightly shortened eye slits, long eyelashes, relatively larger ears, longer philtrum, dimple on the chin, transverse groove on the left palm, and clinodactyly of the V. finger of both hands. The mother and father of the boys are healthy. No other relatives in the family have ASD or a neurodevelopmental condition.

Genetic assessment of the patients included karyotype testing, FMR1 gene triplet repeat expansion analysis, array-based comparative genomic hybridization (aCGH) for CNV detection, and whole exome sequencing. Both boys had normal karyotypes (46, XY). DNA analysis of the 5′ untranslated region of the FMR1 gene did not show an increase in the number of CGG repeats (31 × in A.V. and 33 × in N.V.). CNV analysis utilizing the aCGH method showed heterozygous deletion of 10q26.3 (CHR10: 134427068–135404523, hg19) sized 977 456 bp in both boys. The region includes 28 genes (Table 1).

WES analysis was performed utilizing Whole Exome Solution v2 (Sophia Genetics), covering 21,450 genes. The DNA variants were prioritized based on their reference to the virtual panel for developmental delay and autism, zygosity, mode of inheritance, population frequencies, and pathogenicity predictions. The results of selected DNA variants in both patients are summarized in Table 2. The analysis pointed to the nonsense DNA variant c.2149C > T in the TRIO gene identified in N.V. and the nonsense DNA variant c.159C > A in CLN8 found in both boys.

**Figure 1:**
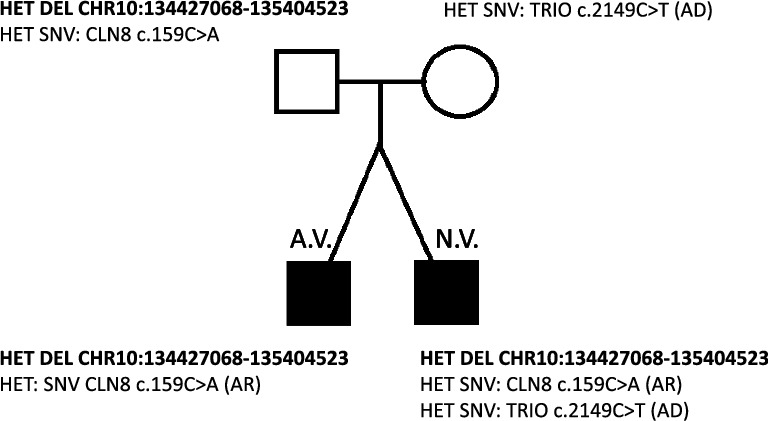
Pedigree of the family with the results of segregation analysis. AR: autosomal recessive, AD: autosomal dominant, HET: heterozygous, DEL: deletion, and SNV: single nucleotide variant. Coordinates of the deletion are shown according to hg19.

The suspected CNV variant and SNV variants with pathogenic or likely pathogenic predictions were subjected to segregation analysis in the family members. The analysis showed that the father of the boys carried the suspected CNV variant in the heterozygous state and the mother of the boys carried the c.2149C > T heterozygous variant in the TRIO gene. The father also carried the c.159C > A in the CLN8 gene (Figure 1). Variants in the ARSA and FH genes were not analyzed in the parents of the probands.

## 3. Discussion

In the present study, both twin boys carried a 1 Mb deletion in the 10q26 region covering 28 genes (Figure 1). Haploinsufficiency in these genes may contribute partially to the phenotypic expression of neurodevelopmental disorder in both boys. The detected deletion is localized in the region affected in the 10q26 deletion syndrome, a phenotypically heterogeneous group of disorders including developmental delay and neurological, cardiovascular, and urogenital tract anomalies (OMIM #609625) [11]. However, the syndrome usually encompasses larger genomic regions ranging from 3 up to 15 Mb, where the size and lost gene content are related to the severity of the phenotype [12]. Another study described four patients with distal deletion of 10q26.3 with atypical presentation of this syndrome including ataxia, mild-to-moderate intellectual disability, and hyperemia of the hands and feet; however, the deletions were of a larger size between 4.6 and 6.4 Mb in length [13]. In our cases, the deletion of 1 Mb length was inherited from the asymptomatic father, meaning that it is not sufficient to cause the neurodevelopmental disease but may contribute to it. In addition, out of the 28 genes located in the deleted region of 10q26, gene SYCE1 belongs to the SFARI gene database as a gene carrying risk for ASD [7]. The gene TUBGCP2 is also of a particular interest, since it is related to the neurological condition of pachygyria, microcephaly, developmental delay, and dysmorphic facies, with or without seizures (OMIM 618737) with an autosomal recessive mode of inheritance. However, WES analysis did not detect any other damaging variants in the remaining copy of this gene in our probands. The incomplete penetrance of the deleted variant points to the search for other DNA variants contributing to the autism phenotype. The WES analysis identified two likely pathogenic SNV variants, of which the variant c.2149C > T in the TRIO gene was present in a heterozygous state only in patient N.V. The TRIO gene follows the autosomal dominant mode of inheritance. The variant is classified by the VarSome database as likely pathogenic; however, it was not previously reported in any patients according to ClinVar and Franklin Genoox databases [10, 14]. The TRIO gene according to the OMIM database is linked to the autosomal dominant intellectual developmental disorder with microcephaly Type 44. TRIO is a strong candidate gene for intellectual developmental disorders. It is highly expressed in the brain during neurodevelopment and is involved in the regulation of neuronal migration, neurite outgrowth, and synaptogenesis [15]. TRIO was described as a negative regulator of neurite outgrowth and synaptic strength [16]. Four variants, protein-truncating mutations, were identified in patients with intellectual developmental disorders, autism, hyperactivity, and/or aggressive behavior [17]. The c.2149C > T variant is present only in patient N.V., who exhibits microcephaly clinically, and is not present in his twin brother, who does not have microcephaly. Barbosa et al. identified nonsense and missense variants in the TRIO gene in 24 patients with intellectual developmental disorders [15]. Carriers of nonsense variants showed a variable neurodevelopmental disorder phenotype, while carriers of missense variants fell into two categories: those with macrocephaly and severe intellectual disability (variants in the spectrin domain of the protein) and those with microcephaly (variants in the RAC1-activating GEFD1 domain of the protein). Impaired activation of RAC1 is associated with microcephaly [15]. Interestingly, one of these patients carried a nonsense mutation c.2302C > T in the spectrin domain, in close proximity to our patient with nonsense variant c.2149C > T. The patient in the study was a female with microcephaly (−3.5 SD), language delay, learning difficulties, and mild Feingold-like dysmorphism [15]. On the other hand, the mother of the twin probands is a carrier of the same nonsense variant without any reported pathology, suggesting that the c.2149C > T variant exhibits an incomplete penetrance.

Both twin boys are carriers of a missense c.159C > A variant in the CLN8 gene. The variant has likely pathogenic clinical predictions according to the VarSome database. The CLN8 gene encodes a transmembrane protein involved in lipid synthesis, transport, or sensing [18]. Mutations in this gene are associated with progressive epilepsy with mental retardation (OMIM#600143), which is a subtype of neuronal ceroid lipofuscinosis with an autosomal recessive mode of inheritance. A nonsense variant in CLN8 in the heterozygous state segregated with ASD in a Japanese family consisting of a father diagnosed with PDD-NOS and three sons diagnosed with Asperger's syndrome [19]. This missense variant is localized 81 bp upstream from the variant c.159C > A, as reported in our probands. However, the variant exhibits incomplete penetrance, since it was inherited from the unaffected father.

A heterozygous pathogenic variant c.851A > G in the ARSA gene was found in the A.V. patient. The gene is associated with metachromatic leukodystrophy, inherited in an autosomal recessive manner [6]. Another pathogenic heterozygous variant in the FH gene was present in the N.V. patient (c.912_918del). Mutations in the gene cause fumarate deficiency with autosomal recessive inheritance [6].

In conclusion, the identified 1 Mb deletion of the 10q26.3 region probably contributed to the phenotype of autism, speech development delay, and mild facial dysmorphia in our probands; however, it was not the single causal genetic factor. A missense c.159C > A variant in the CLN8 gene found in both twin boys may have contributed to the phenotype as well. A nonsense variant c.2149C > T in the TRIO gene with likely pathogenic predictions probably contributed to the phenotypic feature of microcephaly present only in one of the twin boys. Interestingly, the boy carrying this variant had milder ASD core symptom severity than his twin brother. The degree of intellectual disability was not determined, and it was suggested that the boys also differ in this feature with the assumption of a more profound delay in N.V. In addition, the perinatal history of N.V. patient (fetal hypotrophy, IVH) could also be a contributing factor to his more severe neurodevelopmental outcome, in addition to the likely pathogenic TRIO variant. Other genetic factors, including rare DNA variants, e.g., in ARSA, FH, and other genes, and common DNA variants may have contributed to the phenotypic expressions in the boys. We highlight the importance of employing available methods, such as whole exome sequencing alongside micro-aCGH, for the genetic diagnosis of autism patients in Slovakia, as well as advancing unified algorithms to assess the polygenic nature of ASD.

## Figures and Tables

**Table 1:** Genes located in the CNV 10q26.3 (CHR10: 134427068–135404523, hg19) sized 977 456 bp.

Gene	Function (dbGene [5])	OMIM [6]
INPP5A	Intracellular signaling	—
NKX6-2	Transcription factor, development, myelinization	617560
CFAP46	—	—
LINC01166	—	—
LINC01167	—	—
LINC01168	—	—
ADGRA1-AS1	—	—
ADGRA1	Sensory systems, blood pressure, immune responses, food intake regulation, development	—
KNDC1	Dendritic growth inhibition	—
UTF1	Stem cell differentiation	—
VENTX	Transcriptional repressor	—
MIR202HG	—	—
MIR202	Posttranscriptional regulation of gene expression	—
ADAM8	Cell–cell and cell–matrix interactions, including fertilization, muscle development, and neurogenesis	—
TUBGCP2	Brain development and neuron migration	618737
ZNF511	—	—
CALY	Calcium release interacts with the dopamine receptor	—
PRAP1	DNA damage response, cellular signaling	—
FUOM	Fucose metabolism, negative regulation of neuron differentiation	—
ECHS1	Mitochondrial fatty acid beta oxidation	616277
MIR3944	Posttranscriptional regulation of gene expression	—
PAOX	Polyamine metabolism	—
MTG1	Mitochondrial respiration	—
SPRN	—	—
SCART1	—	—
CYP2E1	Cytochrome P450 enzyme, biosynthesis of molecules, metabolism	—
SYCE1^∗^	Meiosis completion	616947, 616950
SPRNP1	—	—

*Note:* Databases used are as follows: dbGene [5], OMIM [6], SFARI gene [7].

^∗^Genes in the SFARI gene database.

**Table 2:** Findings from WES analysis: variants with pathogenic or likely pathogenic predictions according to the VarSome database.

Patient ID	Gene	Chromosome-position (hp38)-ref.>alt. [10]	cDNA	Type of mutation	Zygosity	Db SNP [8]	VarSome [9]	OMIM [6]	Inheritance (OMIM [6])	SFARI [7] gene rank
A.V.	CLN8	chr8-1771213 C > A	c.159C > A	Nonsense	Het	rs780551031	4	607837	AR	2
	ARSA	chr22-50626594 T > C	c.851A > G	Missense	Het	rs199476342	5	607574	AR	—

N.V.	CLN8	chr8-1771213 C > A	c.159C > A	Nonsense	Het	rs780551031	4	607837	AR	2
	TRIO	chr5-14358280 C > T	c.2149C > T	Nonsense	Het	—	4	601893	AD	1
	FH	chr1-241504231 TGACAAAA > T	c.912_918del	Frameshift	Het	rs794727836	5	136850	AR, AD	3

*Note:* Databases used are as follows: Db SNP [8], VarSome [9], OMIM [6], SFARI gene [7], Franklin Genoox [10].
